# A New Symptom

**Published:** 1890-03-01

**Authors:** 


### A NEW SYMPTOM.

Dr. W. B. Hadden writes a paper headed, "A Subjective Sensation in the Mouth of Women" (*Lancet*). This is described as an intolerable burning of the tongue, extending sometimes to the lips and roof of the mouth. All so suffering were nervous, excitable individuals, who flushed readily, varying in age from 39 to 74. The temperature of the mouth was normal. In none of Mr. Hadden's cases, four in number, was there any discoverable reason for this complaint. Mr. Hadden observes that he knows of no references in medical literature to the subject. Benefit was not derived from any form of treatment except in one case, where the local sensation was slightly removed by cocaine tablets. We should imagine this symptom must be classed as an hysterical manifestation.

